# Pathomechanisms of Oxidative Stress in Inflammatory Bowel Disease and Potential Antioxidant Therapies

**DOI:** 10.1155/2017/4535194

**Published:** 2017-06-28

**Authors:** Tian Tian, Ziling Wang, Jinhua Zhang

**Affiliations:** College of Life Science and Bioengineering, Beijing Jiaotong University, Beijing 100044, China

## Abstract

Inflammatory bowel disease (IBD) is a chronic gastrointestinal disease whose incidence has risen worldwide in recent years. Accumulating evidence shows that oxidative stress plays an essential role in the pathogenesis and progression of IBD. This review highlights the generation of reactive oxygen species (ROS) and antioxidant defense mechanisms in the gastrointestinal (GI) tract, the involvement of oxidative stress signaling in the initiation and progression of IBD and its relationships with genetic susceptibility and the mucosal immune response. In addition, potential therapeutic strategies for IBD that target oxidative stress signaling are reviewed and discussed. Though substantial progress has been made in understanding the role of oxidative stress in IBD in humans and experimental animals, the underlying mechanisms are still not well defined. Thus, further studies are needed to validate how oxidative stress signaling is involved in and contributes to the development of IBD.

## 1. Introduction

Inflammatory bowel disease (IBD) is an incurable chronic inflammatory intestinal disorder of the gastrointestinal (GI) tract that dramatically impacts quality of life. Crohn's disease (CD) and ulcerative colitis (UC) are the principal types of IBD. CD may occur in any region of the GI tract involving the ileum and colon in a discontinuous pattern by transmural inflammation, while UC affects only the colon and rectum continuously and is restricted to the mucosa [1]. In clinical situations, CD can be associated with intestinal granulomas, strictures and fistulas, whereas these are not found in UC. In the US, IBD is the second most common inflammatory disorder and mainly affects people 15–30 years of age [2]. The incidence and prevalence of IBD are increasing rapidly worldwide, and the situation in Asia is more severe than that in the West [3, 4]. Thus, IBD is becoming a major global public health problem [5, 6]. Although it is widely known that IBD is an inappropriate immune response to environmental changes and the intestinal microbiota in a genetically susceptible background, the underlying mechanisms remain elusive. Accumulating data from both experimental models and clinical studies indicate that oxidative stress signaling is involved in and contributes to the development of IBD through multiple levels of function. Oxidative stress leads to damages of the mucosal layer in the GI tract and bacterial invasion, which in turn stimulates the immune response and initiates IBD [7]. In this review, we provide an overview of recent studies on oxidative stress and its implications in IBD and the crosstalk between oxidative stress signaling and the host response in the GI tract. Moreover, the therapeutic strategies targeting redox balance and potential molecular mechanisms are also discussed.

Over the past two decades, the understanding of IBD pathomechanisms has progressed rapidly. However, the current IBD treatments are less than ideal because of their significant risks and side effects [8]. More thorough insights into the role of oxidative stress in IBD will absolutely shed light on how to improve IBD therapy, particularly with regard to a combination medication plan that includes the use of natural and synthetic antioxidant compounds. In this review, we sequentially discuss the following: the triggers of oxidative stress, the antioxidant defense system, and the pathomechanism by which oxidative stress together with susceptibility genes, epithelial cells, mucosal immune cells, the microbiota, and environmental factors causes IBD; oxidative stress-related signaling pathways; and future strategies for the development of new antioxidant therapies for IBD patients.

## 2. Oxidative Stress Signaling in the Gastrointestinal Tract

Oxygen metabolism, which is necessary for mammalian cell survival, produces reactive oxygen species (ROS). ROS primarily refer to free radicals such as superoxide (O_2_·¯), hydroxyl radicals (HO·), peroxyl (RO_2_·), alcoxyl (RO·), and hydroperoxyl (HO_2_·); lipid hydroperoxides; and reactive nonradical compounds including singlet oxygen (O_2_), hydrogen peroxide (H_2_O_2_), hypochlorous acid (HOCl), chloramines (RNHCl), and ozone (O_3_) [9]. Reactive nitrogen species (RNS) mainly consist of nitric oxide (·NO), nitrogen dioxide (·NO_2_), nonradical compounds, peroxynitrite (ONOO¯), and dinitrogen trioxide (N_2_O_3_). ROS and RNS are both predominant mediators responsible for the intracellular damages of carbohydrates, proteins, lipids, and nucleic acids and are highly reactive due to their unstable conditions with unpaired electrons. It has been reported that ROS and RNS upregulate the expression of genes involved in innate and adaptive immune responses in the GI tract [10, 11].

Endogenous ROS are principally produced in intracellular organelles, such as the endoplasmic reticulum, mitochondria, peroxisomes, and the nucleus as well as in the cytosol and extracellular matrix, and the mitochondrial electron transport chain is responsible for a large proportion of ROS generation [12, 13]. Mitochondria are the central organelles in energy metabolism and are exposed to ROS. Excess ROS generation leads to lower ATP production, suppression of the intracellular electron transport chain, and DNA damage in mitochondria. If this situation continues, mitochondrial bioenergetics and homeostasis are affected, resulting in cell death [14, 15]. Mitochondria are the primary targets of oxidative stress, though the underlying mechanism of how this leads to IBD has not yet been revealed.

Multiple enzymes, such as peroxidases, NADPH oxidase (NOX), xanthine oxidase (XO), lipoxygenases (LOXs), glucose oxidase, myeloperoxidase (MPO), nitric oxide synthase (NOS), and cyclooxygenases (COXs), participate in endogenous ROS generation by catalyzing chemical reactions [16, 17]. Among these enzymes, mucosal NOXs, such as the NOX2 complex, NOX1, and dual oxidase 2 (DUOX2), have been reported as novel IBD risk factors, further demonstrating that an imbalance in redox homeostasis is critical for IBD pathogenesis [18]. XO mainly functions in the intestinal mucosa, generating ROS and leading to GI tract injuries [19], while MPO is active in inflamed mucosa in UC patients and contributes to the progression of malignancies [20]. NOS have contradictory effects, maintaining GI mucosal integrity as well as causing injuries in UC and peptic ulcers [21, 22]. COX-1 and COX-2 are both upregulated in human ulcers, while COX-2 is closely associated with precancerous alterations in the GI mucosa in colon inflammation and *Helicobacter pylori*-induced gastritis [23].

In addition, oxidative stress can also be activated by environmental factors, such as radiation, chemotherapy, cigarette smoking, luminal antigens, alcohol, drugs, and xenobiotics, all of which contribute to IBD [7]. Iron (Fe) and copper (Cu) in the diet, trans-fatty acids in some types of processed food, and acrylamide in snacks and crackers all trigger oxidative stress by increasing ROS generation [24–26]. High concentrations of alcohol can directly impair the mucosal barrier of the GI tract. Beyond that, various drugs and xenobiotics have been reported to enhance the production of free radicals in the gut [27].

Under physiological conditions, the cell can tolerate a certain level of ROS due to its antioxidant capacity, which is critical for intestinal homeostasis. However, an excessive oxidant load resulting from increased ROS generation or decreased reduction reactions can overwhelmingly enhance membrane permeability, alter the inflammatory response, and result in lipid and protein modifications, DNA damage, apoptosis, and carcinogenesis [28–30]. This condition is known as oxidative stress, a prominent imbalance between the pro-oxidants and antioxidants observed in many human diseases, such as cancer, diabetes, cardiovascular diseases, atherosclerosis, and chronic obstructive pulmonary disease [31]. Oxidative stress has been implicated in autoimmune disorders (rheumatoid arthritis, systemic lupus erythematosus, psoriasis, and celiac disease) and neurodegenerative diseases (Alzheimer's disease, Parkinson's disease, amyotrophic lateral sclerosis, and multiple sclerosis) [31]. Oxidative stress has also been shown to specifically induce gastroduodenal ulcers [32], IBD [33] (the focus of our review), and even gastric and colorectal cancer [34].

## 3. Antioxidant Defenses in the Gastrointestinal Tract

Although uncontrolled oxidative stress is destructive to the GI tract, the body's antioxidant defenses can counteract the effects caused by excess ROS. These defense mechanisms ensure that the concentrations of ROS/RNS are under control and will not exert harmful effects. The endogenous antioxidant system mainly consists of intracellular enzymatic antioxidants, such as superoxide dismutases (SODs), glutathione peroxidase (GPX), and catalase (CAT); intracellular nonenzymatic antioxidant glutathione; and extracellular antioxidants including vitamins, minerals, ceruloplasmin, and uric acid.

### 3.1. Intracellular Enzymatic Antioxidants

SODs, metal ion cofactor-requiring enzymes, can catalyze the reduction of O_2_·¯ into O_2_ and further into H_2_O_2_. Three SOD isoforms exist in mammalian cells: SOD1 (Cu/ZnSOD, cytosol), SOD2 (MnSOD, mitochondria), and SOD3 (Cu/ZnSOD, extracellular) [35]. As the major form of SODs, SOD1 accounts for almost 70% of the SODs in the cell and resides in the cytoplasm, where it catalyzes the dismutation of O_2_·¯ into H_2_O_2_, and SOD1 or catalase can subsequently remove uncharged H_2_O_2_ from the mitochondria into the cytosol. SOD2 protects the mitochondria from O_2_·¯ and is essential for cell survival, as SOD2-null mice do not survive after birth [36]. SOD3 is prone to binding to glycosaminoglycans such as heparin [37]. Clinical data have demonstrated differential mucosal expression of three SOD isoforms in IBD patients: SOD2 expression was dramatically upregulated, while SOD1 was less affected, and SOD3 was decreased particularly in intestinal epithelial cells (IECs) [38]. SOD activity is associated with the disease course of IBD patients [39]. SOD activity induced by intrarectal acetic acid is also found higher in both UC and CD patients than in controls, and elevated SOD levels can be restored to normal when patients are in remission [40, 41]. On the whole, the total activities of SODs are increased in IBD pathogenesis as a reaction to protect the tissue against oxidative damage under the condition of inflammation and oxidative stress in IBD. Consistent with this, SOD levels in the peripheral blood of IBD patients are already being used as a biomarker for oxidative stress.

GPX can catalyze glutathione into oxidized glutathione (glutathione disulfide (GSSG)) and reduce H_2_O_2_ into H_2_O or lipid hydroperoxides (ROOH) into stable alcohols. GPX, together with glutathione reductase (GSSG-R), maintains low glutathione (GSH) levels and protects the cell from peroxide damage. There are eight GPX isoforms in humans, and most of their active sites contain selenocysteine residues [42]. GPX2 is specifically expressed in the epithelium of the GI tract, while GPX1 is ubiquitously distributed. GPX2 plays a major role in defending against oxidative stress and inflammation in the intestinal mucosa in mouse models of UC and CD and has been reported to be induced in pathogenic conditions such as gastric cancer and IBD [42–44]. Increased GPX2 expression has also been shown in colitis, which is induced by signal transducers and activators of transcription (STAT) factors [45, 46]. The combined mutations of GPX1 and GPX2 in mice result in symptoms resembling IBD due to oxidative damage and inflammation in the GI tract [47]. In addition, GPX3 secreted by IECs contributes to the extracellular antioxidant activities in the intestinal mucosa [48]. Actually, there are many controversial and conflicting reports in comparing the antioxidant capacities of SOD and GPX in IBD with their respective controls due to diverse experimental methods and conditions: therefore, additional researches are necessary.

CAT is mainly located in peroxisomes and catalyzes the reduction of H_2_O_2_ into H_2_O and O_2_ [49]. It is primarily distributed in the human liver, kidney, and erythrocytes. Altered CAT expression can be caused by a series of pathogens related to GI tract diseases, such as *Escherichia coli*, *Shigella*, *Salmonella*, *Campylobacter jejuni* [50], *Helicobacter pylori* [51], and *H. hepaticus* [52]. Decreased CAT activity has been reported in patients affected by CD [53], colorectal cancer [54], gastric adenocarcinoma, and *H. pylori*-positive stomach ulcers [55]. Experiments in genetically modified mice demonstrated that enhanced CAT activity is associated with reduced occurrence of colitis and colon cancer [56, 57].

### 3.2. Intracellular Nonenzymatic Antioxidants

Glutathione is a significant intracellular nonenzymatic antioxidant. Its reduced form, GSH, a more prevalent agent, is a soluble antioxidant that is highly expressed in the cytoplasm, nucleus, and mitochondria. GSH, together with three related enzymes, glutathione peroxidase (GPX), glutathione reductase (GSR), and glutathione S-transferases (GST), forms an antioxidant barrier in the gut mucosa. GSH has been utilized as a biomarker for both inflammation and oxidative stress. In some cases of inflammation, GSH consumption increased sharply [58], while in different IBD subtypes, the GSH level exhibited contradictory trends. Experimental colitis models normally show decreased GSH levels [59–61]. A lower GSH level has been observed in dextran sulfate sodium- (DSS-) induced colitis and can be restored to a normal level by antioxidants [47, 62].

*Melatonin* (MEL) is a strong antioxidant that is originally synthesized in the pineal gland in mammals. It can reduce oxidative stress in both lipid and aqueous cell environments by crossing physiological barriers, such as the mitochondria membrane [63, 64]. MEL scavenges peroxyl and hydroxyl radicals and plays a protective role in early and advanced stages of several diseases involving ROS metabolites including IBD [65–67]. We will discuss the detailed potential of MEL as a medication in the section on IBD therapies.

### 3.3. Extracellular Antioxidants

Vitamin A or *β*-carotene (pro-vitamin A) is found in yellow fruits and green vegetables and exhibits remarkable antioxidant activity that is dependent on retinol-binding proteins. Vitamin C synthesized from glucose can be obtained from fresh fruits and vegetables and can reduce various ROS. Vitamin E is reported to protect the cell membrane from lipid peroxidation. These vitamins are all antioxidants that have been reported to be insufficient in the blood of many UC or CD patients mostly due to the lack of fruits and vegetables in their diets [68–70].

Minerals including zinc (Zn), copper (Cu), manganese (Mn), and iron (Fe) are essential for some antioxidant enzymes. Different isoforms of SOD require Zn, Cu, or Mn as cofactors, and iron is needed for CAT activity [71]. Polyphenols are mainly obtained from plants. Flavonoids, important polyphenols, suppress XO and other ROS-related enzymes, such as COX, LOX, GST, and NOX [72, 73]. These extracellular antioxidants mainly exist in fruits and vegetables.

## 4. Pathomechanisms of Oxidative Stress in IBD

Although the mechanisms underlying the etiology of IBD have not been thoroughly illuminated, it is commonly accepted that numerous factors including genetic susceptibility, alterations in IECs, dysregulation of immune responses, intolerance to the microbiota, and environmental factors in a background of oxidative stress together contribute to IBD development.

### 4.1. Genetic Susceptibility in IBD and Oxidative Stress

In recent years, the genetic variations of IBD patients have been comprehensively investigated. With the rapid development of new high-throughput technologies and large-scale collaborations worldwide, researchers have found 200 genetic risk loci associated with IBD, including *NOD2/CARD15*, *ATG16L1*, *IRGM*, *CARD9*, *RNF186*, *PRDM1*, *IL-23R*, and *IL-10* [74–76].

In addition to the reported disease-specific loci, UC and CD share some susceptibility genes, such as *IR23*, *IL12B*, *NKX2-3*, and *MST1*. The UC-specific risk loci are *IL-10* and *HLA*, while the CD-specific ones are NOD2, ATG16L1, and IRGM1 [77–81]. In CD, NOD2/CARD15 is the most important locus, and it can affect the mucosal recognition of bacteria and contribute to the duration of nuclear factor kappa B (NF-*κ*B) activation and inflammation [82]. Genome-wide association studies (GWAS) have also identified other genes that are functionally related to immunodeficiency, such as RIPK2, TNFSF15, IFNGR1/2, TYK2, and CARD9 [83–85]. Autophagy, which is activated to digest cellular organelles upon cell starvation or infection, has been recognized as a pathogenic mechanism of CD. For instance, the variant T300A of ATG16L1 has been widely reported as a risk factor that functions in autophagy during CD-related intestinal inflammation [75, 86], which also facilitated the identification of susceptibility genes IRGM1, SMURF1, and ATG16L2 in CD [83, 87–89]. Mutations in *NOD2* and *ATGL16L1* have been found to contribute to morphological alterations in Paneth cells in CD patients [90]. In addition, NOD2, ATG16L1, and IRGM1 have been suggested to interact with each other [76]. Mutations in *immunity-associated guanosine triphosphatase M* and *leucine-rich repeat kinase 2*, which are essential in autophagy, and variations in *NLRP3* have been reported to be related to CD [91–93]. Functional analyses of these susceptibility genes and their related pathways all highlighted the defective innate immunity present in CD. *HLA class II* genes, *cadherin*, *lamininβ1*, and *MUC19* have been discovered to be associated with UC in a GWAS meta-analysis [81, 94]. Caucasian CD and UC populations shared the mutation D299G in Toll-like receptors 4 (TLR4) [95], while variants of IL-23R and IL-17A have been found in subsets of Korean UC and CD populations [96, 97]. Along with the IL-23R pathway, the susceptibility genes *IL-12B*, *JAK2*, and *SATA3* have also been discovered in CD [98], whereas polymorphisms in *IL-22* and *IL-10 receptor 2* have been found, respectively, in UC patients and children with early-onset enterocolitis [79, 99]. The functions of susceptibility loci identified by GWAS analyses have not been fully deciphered, and further detailed genetic studies are still needed. These susceptibility genes also explain why the family histories in both CD and UC patients and their relatives are more common.

Variations of antioxidant/biotransformation enzyme genes can alter the enzyme activity and contribute to the risk of IBD. For example, the polymorphisms *NAD(P)H:quinone oxidoreductase 1* (NQO1) C609T and *SOD2* Ala-9Val are involved in the risk of UC and clinical responses to therapies [100]. Mutations of GST M1 or T1 are related to IBD progression, and the two combined will further increase the risk. UC patients with defective GST M1 activity in the whole blood exhibited early disease onset and more severe symptoms [101, 102]. The paraoxonase (PON) gene located on chromosome 7q21.3-22.1 shows characteristics of a susceptibility gene in both UC and CD. In a case-control study, the elevated PON1 activity of the PON1 R192 allele protected against IBD development in a Jewish population from Israel [103]. In addition, a polymorphism in the promoter of *Nrf2*, whose protein product regulates the expression of intestinal detoxifying and antioxidant genes and acts as a defender, has been associated with UC development in a Japanese population [104].

### 4.2. Epithelial Cells of the Gut in IBD and Oxidative Stress

The intestinal epithelium, which exhibits both organ-specific and immune functions, has been recognized as playing a central role in the pathogenesis of IBD. Epithelial cells form a cell barrier lining the GI tract between the host and various organisms, which is essential for maintaining mucosal homeostasis. The barrier layer is maintained by tight junctions, adherens junctions, and desmosomes. Paneth cells, goblet cells, absorptive cells, and enteroendocrine cells are the four major secretory cell types in IEC populations [105], and Paneth cells play an essential role in controlling bacteria via the secretion of antimicrobial peptides [106]. IECs express pattern-recognition receptors (PRRs), such as TLRs, to distinguish commensal and invasive pathogenic bacteria, which are important for intestinal mucosal homeostasis [107, 108]. Once excessive ROS are generated in the gut, oxidative stress can accelerate cell damages by modifying the functions of proteins and causing lipid peroxidation [109]. During mucosal inflammation, IECs as well as neutrophils and macrophages produce superoxide and nitric oxide by activating NOX and inducible nitric oxide synthase (iNOS), respectively, both induced by inflammatory cytokines. IECs produce more ROS/RNS via NOX and iNOS activation. This ROS overload can damage cytoskeleton proteins and lead to alterations in tight junctions and epithelial permeability in IECs, finally resulting in barrier disruption [110, 111]. In this way, oxidative stress inflames the GI tract, and IBD is initiated. During inflammation, bacteria can penetrate the epithelial barrier and activate PRR signaling at the endothelium, elevating TLRs and NOD expression on endothelial cells [112]. Endothelial cells also express CD40 and CD40L proteins, which belong to the tumor necrosis factor (TNF) receptor superfamily and can costimulate immune cells during inflammation [113]. All of the above demonstrate that the endothelium acts as a second barrier during inflammation. The microvasculature surrounding the epithelial cells can recruit inflammatory mediators, leading to further tissue damages and inflammation expansion. This inflammation finally causes mucosal injuries accompanied by alterations in IECs, such as goblet cell loss, crypt cell hyperplasia, reduced mucous production and ulcerations.

### 4.3. Mucosal Immune Cells in IBD and Oxidative Stress

In the GI tract, innate immunity consists of epithelial cells, neutrophils, macrophages, dendritic cells, and natural killer (NK) cells, while the adaptive immune system consists of T lymphocytes and B cells, which release cytokines and antibodies upon activation. The mucosal immune system can delicately distinguish pathogenic, nonpathogenic, and normal gut microorganisms via an efficient mechanism. Under normal conditions, the balance between proinflammatory (TNF-*α*, IL-1, IL-6, IL-8, IL-17, and IL-23) and anti-inflammatory (IL-5, IL-10, IL-11, and TGF-*β*) cytokines is tightly controlled in the GI mucosa. Neutrophils together with other cell types initiate inflammation by secreting proinflammatory cytokines to further activate the adaptive immune system [114]. Recently, it has been accepted that IECs also participate in the adaptive immune response [115].

Innate and adaptive immunity are both affected in IBD, while adaptive immunity can sustain inflammation in CD but not initiate it [116, 117]. The immune mechanism underlying IBD pathogenesis is a disruption of the balance between T helper (Th) cells and regulatory T cells, particularly the intolerance of regulatory T cells. CD is reportedly characterized by Th1 cell-mediated inflammation with excess IL-12, IL-17, and IL-23 production, whereas UC is dominated by cytokines such as IL-4, IL-5, IL-10, and IL-13 produced by Th2-type T cells [76]. In CD, the microbiota triggers the Th1 response, generating interferon-*γ* and TNF-*α* and leading to the mucosal barrier damage [118]. The NF-*κ*B signaling pathway is activated not only in leukocytes but also in the mucosal IECs of CD patients [119]. In UC, NK cells generate Th2 cytokines, arousing the immune response, and IL-13 mainly leads to IEC apoptosis, which provides positive feedback to NK cells [120, 121]. The activation of immune cells, mainly macrophages and neutrophils, can cause superoxide and nitric oxide generation by NOX and NOS and the production of the oxidant peroxynitrite, all of which are involved in the pathogenesis of IBD and the initiation and progression of CD in particular [45, 122, 123]. During inflammation, other immune cells, such as leukocytes and monocytes, can also augment ROS production during respiration, prostaglandin, and leukotriene metabolism, resulting in further tissue damage [124].

In mouse colitis models, the loss of macrophages or neutrophils decreases ROS/RNS production, reduces proinflammatory cytokine generation, and alleviates intestinal inflammation and damage [125]. The active neutrophils and macrophages as well as the IECs in the GI tract generate more ROS, which can be inferred by the level of 8-hydroxy-2′-deoxyguanosine (8-OHdG) in the blood, a biomarker for oxidative damage in IBD patients [76]. The infiltration of inflammatory cells in the mucosal further damages the GI tissue, forming a vicious cycle.

### 4.4. Interaction between Oxidative Stress and the Microbiota in the GI Tract in IBD

The human gut microbiota includes the population of microorganisms, such as bacteria, viruses, and fungi that inhabit the GI tract. The microbiota is involved in several protective, structural, and metabolic functions and plays a pivotal role in gut homeostasis and host health. Microbiota restricts the proliferation of pathogenic bacteria in the GI tract, activates the immune system, regulates nutrient utilization and host metabolism [126, 127], and controls vitamin and enzyme production. The microbiota also produces short-chain fatty acids (SCFAs), ethanol, lactate, phenols, and succinate; degrades proteins and carbohydrates; and transforms bile acids [128]. In particular, SCFAs derived from bacteria fermentation, including butytrate, propionate, and acetate, are essential for mucosa homeostasis and anti-inflammatory interleukin production [129]. Previous evidence has demonstrated that the balance between the commensal microbiota in the GI tract and host mucosal immune responses plays a significant role in the pathogenesis and duration of IBD [130]. Several commensal microorganisms are suspected to function as potential pathogenic factors in the development of IBD, such as *E. coli*, *Saccharomyces cerevisiae*, and *Mycobacterium avium paratuberculosis* (MAP). In UC, mucosa-associated *E. coli* was found in 68% of patients compared with 6% of controls, suggesting that it plays a possible pathogenic role [131]. MAP has been investigated for its latent function in CD pathogenesis, which remains controversial [132–134]. Recently, in gut flora research, there has been an emerging trend of studying the entire composition and associated causality of all types of flora in IBD patients. Dysbiosis, the alteration of the commensal microorganism composition or the decreased complexity of the microbiota ecosystem, has been featured in UC and CD patients. IBD treatments also affect the microbiome [5, 135–137]. Thus, studies of the microbiome may inform the development of probiotic-based treatments for IBD in the future.

ROS induction plays a central role in microbiota-linked GI diseases, including IBD and colorectal cancer. ROS are associated with intestinal dysbiosis. During inflammation, the gut microbiota can generate ROS directly, which can also cause DNA damage in infected cells [138–140]. It has been reported that *H. pylori* can induce neutrophils to produce ROS while generating ROS itself, contributing to gastric carcinogenesis [141]. The situation is similar to that in IBD. Gut microorganisms can accelerate NO and NOS production by the host via macrophage activation in response to inflammation inducing DNA damage. Some specific bacteria can produce NOS [142]. Oxidative stress is caused by an imbalance in pro-oxidative molecules and antioxidant defenses, leading to cellular impairments, including DNA damage, protein aggregation, and membrane dysfunction. Upon bacterial infection, the DNA repair system is altered by bacteria modulation. It has been reported that some enteropathogenic *E. coli* strains can affect the DNA mismatch repair (MMR) system by secreting effector proteins in HT-29 cells in vitro. Thus, the accumulation of mutations due to defects in the MMR system can induce inflammation-associated carcinogenesis [143, 144]. Oxidative stress caused by ROS generation stimulates an initial inflammatory response via positive feedback and leads to additional ROS production and further tissue damage [69]. Obviously, more studies are needed to further elucidate the underlying mechanisms involved in the interactions between the gut microbiota composition and oxidative stress in IBD development.

### 4.5. Environmental Factors in IBD and Oxidative Stress

Environmental factors such as sanitary issues, high consumption of saturated fats and refined sugar, frequent use of antibiotics, and even social stress are all suspected to contribute to a high risk of IBD [145]. These factors may be more accurately categorized as lifestyle factors [6, 132, 146, 147]. Here, we mainly focus on two environmental factors in IBD and their association with oxidative stress: smoking and wine consumption. Smoking is the most significant environmental factor for CD risk according to epidemiological data, whereas it positively affects the process of UC development. The underlying mechanism of its dual roles in CD and UC has not yet been clarified [148]. Among more than 4500 chemical components in tobacco smoke, nicotine, carbon monoxide (CO), and NO are the main active modulators that function in the intestinal immune system and are involved in IBD pathogenesis [149]. Several studies have explored the effects of smoking in animal models through exposure to nicotine or other tobacco smoke via oral administration or injection of these ingredients. One area of research focuses on the effects of tobacco smoke on inflammatory cells and mediators, while another concentrates on the intestinal epithelium and mucosal layer. Above all, cigarette smoke can interact with oxidative stress in IBD development. First, in addition to influencing ROS-generation pathways, cigarette smoke also contains a high concentration of ROS in its gas and tar. Second, metal ions in tobacco smoke can facilitate the transformation of hydrogen peroxide into the highly reactive hydroxyl radical [150]. It has been reported that iNOS activity is increased and oxidative stress is elevated in the small intestine after nicotine administration. Cigarette smoke exposure dramatically inactivates SOD in the colon, while the GSH level remains stable, indicating a reduced capacity to remove free radicals in the colon [151–153]. These findings demonstrate that smoke can sensitize the gut to oxidative stress and promote the IBD process.

Wine consumption is another environmental factor that is pervasive in daily life, though its effects on IBD remain controversial. The beneficial aspects of wine are mainly attributed to its most abundant nonalcoholic components, the phenolic compounds, which can protect the intestinal mucosa from oxidative stress as antioxidants [154]. The polyphenols in wine can be digested by IECs or consumed by the gut bacteria, which is important for their bioactivities. These compounds act as scavengers of free radicals or interfere with inflammatory molecules during IBD [155, 156]. The other effects of wine on IBD come from the alcohol, which can lead to prolonged oxidative stress and inflammation, especially at high concentrations. Alcohol can cause mucosal damage directly and even elevate the risk of colorectal cancer [157, 158]. Thus, it is essential to distinguish among the different components in wine and clarify their interactions with oxidative stress in IBD. Generally, moderate wine consumption is recommended for inactive IBD patients as well as healthy people.

### 4.6. Redox Signaling Pathways Involved in IBD

#### 4.6.1. NF-*κ*B Signaling Pathway

NF-*κ*B is a significant regulator in multiple pathogenic processes and is aberrantly activated in IBD [119, 159, 160]. NF-*κ*B can modulate the permeability of the intestinal layer and is associated with IEC homeostasis [161], while chronic NF-*κ*B signaling may also intensify the chronic intestinal inflammation observed in the mucosa of UC and CD patients [119, 159]. Microbial-activated TLRs and TNF-*α* recognized by IECs stimulate downstream NF-*κ*B signaling events, which are also sensitive to oxidative stress. TNF-*α* facilitates superoxide generation in colon cells via the activation of NOX1 and NADPH oxidase organizer 1 (NOXO1) [162]. NOX activation, ROS generation, LC8 peptide release, and I*κ*B*α* phosphorylation are all involved in the activation of NF-*κ*B by TNF*α* [163]. Studies in DSS-induced murine models have demonstrated that phenyl-N-tert-butylnitrone, an antioxidant drug that suppresses NF-*κ*B, can alleviate inflammation symptoms. This finding also proves that oxidative stress contributes to the activation of NF-*κ*B signaling in IBD [164].

NF-*κ*B activation can elevate the transcription of five types of genes that are pivotal in IBD pathogenesis: first, proinflammatory cytokines including IL-6, IL-8, IL-16, and TNF-*α*; second, genes modulating cell survival and propagation, such as the p53-upregulated modulator of apoptosis (PUMA), whose upregulation by NF-*κ*B causes epithelial cell apoptosis and contributes to UC development [165]; third, genes associated with the permeability of the intestinal barrier, for instance, the myosin light chain kinase (MYLK), which is upregulated by TNF-*α*-induced NF-*κ*B activation and leads to the degradation of myosin in the intestinal barrier [165]; fourth, metalloproteinases, which can digest mucosal cells and are also triggered by TNF-*α*; and fifth, enzymes including COX-2 and iNOS, which activate NF-*κ*B and interfere with barrier stability via ROS metabolism [159].

#### 4.6.2. Nuclear Factor-Erythroid 2-Related Factor 2 Signaling Pathway

Nuclear factor-erythroid 2-related factor 2 (Nrf2) is a key transcription factor that can maintain mucosa homeostasis by suppressing excessive ROS generation in IBD. Nrf2 negatively regulates the proinflammatory response and mucosal impairment via an antioxidant mechanism. *Nrf2-*KO mice demonstrated more severe symptoms with a substantial loss of crypts compared with those of wild-type mice in a DSS-induced colitis model. Furthermore, *Nrf2-*KO mice showed increased expressions of proinflammatory genes, including *IL-1β*, *IL-6*, *IL-8*, *iNOS*, and *COX-2*, and decreased expression of antioxidant enzymes, such as hemeoxygenase-1 and GST Mu-1 [166, 167]. The gene *immediate early response-3* (*IER3*) negatively modulates Nrf2 activation through the phosphatidylinositol 3 kinase/protein kinase B (PI3K/Akt) pathway. A lack of *IER3* stimulates Akt and Nrf2 and results in reduced ROS production and apoptosis in a colitis model [168]. Thus, Nrf2 is kept at a low level in IBD because of activated IER3 in the mucosa.

With regard to oxidative stress, Nrf2 has been reported to suppress protein kinase C and decrease NOX activation by increasing the level of the antioxidants GSH, which also explains why ROS levels and production are decreased in *Nrf2*-KO mice [169]. Moreover, upon NOX1 activation, cholesterol oxidation products, oxysterols, contribute to free radical driven-apoptosis and induce the cytokines IL-1*β*, IL-6, IL-8, and IL-23 and monocyte chemotactic protein-1 (MCP-1) as well as TLR2 and TLR9. In this way, oxysterols promote mucosal barrier damage in IBD [170, 171].

As discussed in previous sections, intracellular enzymatic antioxidants such as MnSOD, Cu/ZnSOD, GPX, and CAT are upregulated in the mucosa of UC and CD patients [38, 44, 172]. In a mouse model of severe acute colitis, overexpression of *Cu/ZnSOD* decreased mortality but had no protective effects on chronic inflammation of the GI tract [173]. A murine colitis model showed that *Gpx2* is upregulated via signal transducer and activator of transcription (STAT) not Rrf2 [46]. In addition, spontaneous ileocolitis developed in *Gpx1* and *Gpx2* double knockout mice due to *Nox1* overexpression and excessive ROS production [47, 174, 175]. Experimental results in vitro indicate that GPX2 inhibits COX-2 expression and membrane-associated prostaglandin synthase-1 (mPGES-1) in the intestine while detaching prostaglandin E2 (PGE2) [176].

Furthermore, oxidative stress will cause DNA damage primarily in the mitochondria. The instability of mitochondrial DNA contributes to increased susceptibility to colorectal cancer transformed from UC [12, 177]. Conversely, treatments with antioxidant ubiquinone that targets mitochondria, MitoQ, can decrease mitochondrial ROS and relieve the symptoms of colitis in a DSS-induced mouse model [178]. Mitochondria are regarded as significant cellular drivers and mediators of the inflammatory process [179]. The roles of mitochondria in oxidative stress require further exploration, which may lead to a better understanding of IBD initiation and progression.

## 5. New Therapies Targeting Oxidative Stress in IBD

IBD is a typical intestinal disorder that involves dysfunctions in immune response. The leading ideology of IBD treatments mainly focuses on reducing inflammation. In traditional therapeutic approaches, anti-inflammatory agents, such as sulfasalazine, mesalazine, infliximab, and corticosteroids, are utilized to antagonize inflammation and promptly relieve IBD symptoms. These agents exert their functions by blocking TNF-*α*- or NF-*κ*B-mediated inflammation. Nevertheless, these anti-inflammatory agents often cause side effects, such as Gl problems, anemia, and hypersensitivity, when taken for the prolonged treatments of IBD and may result in drug intolerance. Immune modulators, such as thiopurines and cyclosporine, are alternative choices that can be used to treat IBD via immunosuppression [118, 180]. These drugs are commonly used together with anti-inflammatory agents, though immune modulators can have cytotoxic effects that may enhance other diseases or infections. Some of these drugs have direct free radical-scavenging capabilities, while others have indirect antioxidant functions via TNF-*α*.

Thus, the use of these drugs must be carefully monitored as part of a personalized medication plan [181, 182].

As the incidence and severity of IBD and colorectal cancer have increased, demands have grown for revolutionary therapeutic approaches for IBD. Because the pathomechanism of oxidative stress in IBD has been suggested by many laboratory reports and clinical trials, regulating Nrf2 signaling and suppressing ROS generation by targeting NOX or mitochondria are both potential treatment options for IBD. Furthermore, some nonconventional therapeutic methods with antioxidant effects, such as ROS generation inhibitors, hormones, functional dietary interventions, and natural or synthetic substances that inhibit cell death and activate antioxidant enzymes, have attracted increasing attentions as complementary and alternative medicines (CAMs) for IBD [183, 184]. These antioxidant therapies may have fewer side effects, lower costs, and better treatment responses, offering new hopes to IBD patients, especially those with UC in both the active phase and the remission stage of the disease [185]. As we discussed above in the antioxidant defense section, intracellular or extracellular enzymatic or nonenzymatic antioxidants could be developed into potential medications for IBD.

### 5.1. Inhibitors against ROS Generation

Drugs that have antioxidant effects primarily include COX-2 inhibitors, angiotensin II type 1 inhibitors, and hydroxymethylglutaryl CoA reductase inhibitors. Molecules that can block the activity of enzymes (such as COX-2 inhibitors) have been reported to reduce endogenous ROS generation and inflammatory responses in IBD patients [186]. Among the candidate angiotensin II type 1 blockers, telmisartan (TLM) has both anti-inflammatory and antioxidant characteristics and exhibits protection in the treatment of colitis. It has been reported that oral TLM administration inhibits ROS production by suppressing NF-*κ*B, COX2, and iNOS expression in a rat model of colitis. In addition, TLM can suppress lipid peroxidation, remove NO·, and increase GSH levels and SOD, GPX activities in this model, all of which contribute to oxidative stress reduction [187]. Inhibitors of hydroxymethylglutaryl CoA reductases are often referred to as statins, such as simvastatin and rosuvastatin, which attenuate oxidative stress by inhibiting MPO activity, decreasing lipid peroxidation, and increasing SOD activity, as reported in a rat model [188].

### 5.2. Hormones

MEL has been successfully tested as a nontraditional hormonal therapy for IBD. Generally, MEL functions directly on the GI tract in an endocrine, paracrine, or autocrine manner to influence the epithelium and regulate the gut environments [189]. Several experimental and clinical reports have verified the protective roles of MEL as an inflammation regulator, as discussed in the antioxidant defense section of this review. MEL acts as a specific antioxidant that scavenges free radicals, leading to decreased oxidative damages in either lipid or aqueous cellular environments during both the initial and advanced phases of IBD [65, 190]. MEL also acts as an antioxidant indirectly by modulating antioxidant enzymes, such as SOD and GPX [191, 192]. It has multiple potential targets such as Nrf2, STAT3, IL-17, sirtuins, poly (ADP-ribose) polymerase 1 (PARP1), and death proteins and is involved in telomere dysfunction and epigenetic alterations: these functions could be used to design novel strategies to alleviate the symptoms of UC. The dose and administration time of MEL are key factors in the treatments of UC patients [193]. In addition, MEL suppresses autophagy induced by ROS and sirtuins, both of which correlate with the circadian clock machinery and might contribute to antitumorigenesis in the colon [194]. MEL metabolism is altered in UC patients and is associated with disease severity, indicating that MEL supplementation may help relieve symptoms in UC patients [195]. MEL also regulates the intestinal immune system in colitis models. It could be introduced as a supplemental medication in IBD treatments, though its side effects over the long term have not been determined. Additional clinical trials are still needed to determine the antioxidant effects of MEL.

### 5.3. Synthetic Substances

N-acetylcysteine (NAC) is a synthetic substance that provides cysteine for GSH synthesis and can increase the antioxidant defense activity, showing benefits in both animal model experiments and clinical tests in IBD patients [196, 197]. NAC can scavenge the free radicals and metals involved in ROS generation and the inflammatory responses [198]. Moreover, NAC inhibits NF-*κ*B, and its antioxidant effects contribute to the recovery of IBD [199]. In mouse IBD models, oral treatments with NAC for one and a half months led to decreased lipid and protein oxidation, increased GSH and CAT activities, and improved antioxidant status in the colon, demonstrating that long-term oral administration may be more beneficial for patients [200].

Modified SOD and propionyl-L-carnitine (PLC) have also been applied in clinical trials because of their strong treatment effects in IBD [201, 202]. Intravenous administration of a lecithinized human Cu/ZnSOD (PC-SOD) was found to suppress MPO and NF-*κ*B activity. PC-SOD also exhibited benefits and safety when administered to UC patients for 14 days [203, 204]. In addition, a recombinant SOD (rec-SOD) elevated antioxidant activity and reduced ROS damage as well as MPO activity in the colon of a mouse colitis model [202]. PLC, an antioxidant molecule, can suppress ROS generation and remove free radicals leading to symptom relief in UC patients in phase II clinical trials [205, 206]. Further studies are needed to determine the optimal safe dose of PLC and the effects of its long-term use.

### 5.4. Polyphenols and Other Herbal Plant Extracts

Polyphenols are a class of over 7000 compounds that are abundant in dietary fruits and vegetables with high fiber content. Polyphenols have antioxidant, anti-inflammatory, immunomodulatory, and apoptotic characteristics and can regulate several target genes involved in inflammation, such as NF-*κ*B, Nrf-2, STAT, and mitogen-activated protein kinases (MAPKs). Polyphenols also inhibit the generation of cytokines, such as IL-8, IL-1*β*, and TNF-*α*, and promote the activities of intracellular antioxidants including SOD and GPX. Through their antioxidant activities, polyphenols directly scavenge free radicals and interrupt some redox signaling pathways, such as the NF-*κ*B and MAPK pathways [156, 207]. In addition, polyphenols protect the intestinal mucosal by reducing intestinal permeability via tight junction stabilization. They also enhance the healthy microbiota in the gut and the production of SCFAs similar to probiotics [208]. A systematic review of epidemiological observations showed that a lower risk of both CD and UC is associated with a high intake of fruits and vegetables. Clinical trials of IBD interventions have demonstrated that 22 CD patients who followed a semi-vegetarian diet rich in polyphenols for over 2 years reported symptom relief [209]. Another study from a murine colitis model showed that polyphenols can decrease the harmful effects of ROS on the intestinal mucosa. However, attention should be paid to the following negative effects of polyphenols: first, they may act as pro-oxidants at high doses; second, they may affect the absorption of other drugs; and third, they may interfere with metabolism pathways.

Polyphenols can be classified as flavonoids (such as isoflavonoids and anthocyanins) and nonflavonoids (such as phenolic acids, coumarins, stilbenes, and tannins). Flavonoids have been reported to protect the cell against oxidative stress and preserve the epithelial mucosa layer of the gut in vivo and in vitro [210]. Resveratrol from grapes, berries and peanuts, curcumin from *Curcuma longa*, quercetin from *Quercus* species, catechin from green tea, and proanthocyanidins from grape seeds and berries all represent polyphenols with antioxidant capabilities. Anthocyanins are an important subset of flavonoids and are ubiquitously distributed in many berries, such as strawberries, blueberries, cranberries, blackberries, and sunrouge buds. Anthocyanins play therapeutic and protective roles in the management of IBD by inducing anti-inflammatory cytokines, modulating TLR4, and improving the intestinal microbiota [211]. Another polyphenol, curcumin, a yellow agent derived from turmeric, has long been used in traditional medicines to treat inflammation and chronic diseases [212, 213]. Curcumin can reportedly scavenge free radicals and decrease oxidative stress and inflammation via the Nrf2 signaling pathway or can act as a TNF blocker in vivo and in vitro [214–216]. In a rat model, oral administration of curcumin alleviated colon damage and decreased inflammation and apoptosis [217]. Several clinical studies have reported the benefits of curcumin in IBD patients without serious side effects [218–221]. Therefore, curcumin is a promising drug for IBD treatments.

There are also other antioxidant substances extracted from natural plants or herbal medicines that may be useful in the design of IBD medications. To date, 55 herbal or plant products have been studied in various IBD models, including thyme, lemongrass, ginger extract, *Panax notoginseng*, and guggulsterone, some of which have long been utilized in Chinese or Indian traditional medicine for IBD treatments [222]. However, some of these compounds may also exhibit toxicity problems due to their pro-oxidative properties, leading to liver and renal damage [223]. Thus, further trials of herbal extracts in animal models are indispensable. So far, no herbal extracts from plants have been certified as adjuvant medications in IBD by the International Society of Nutritional Therapy [224, 225].

### 5.5. Functional Dietary and Antioxidant Micronutrients

Functional foods for IBD therapy may include food in a diet, such as camel's milk, or an ingredient added to a diet, such as extra virgin olive oil (EVOO). Camel's milk is rich in many minerals (Ca, Fe, Mg, Cu, and Zn), vitamins (A, B2, C, and E), insulin, and lactoferrin, with excellent antioxidant and anti-inflammatory effects, and it is deficient in sugar, protein, and fat. It has been used as a nutritional supplement for immune-deficient patients and may become a valuable replenishment for IBD patients [226]. EVOO consumption can activate antioxidant enzymes such as CAT, SOD, and GPX and regulate immune responses against oxidative stress to relieve IBD symptoms [227]. EVOO is widely reported to protect against colon cancer, which is a later stage of IBD progression [228].

Moreover, many types of nutrients are found to act as antioxidants in IBD, such as glutathione; vitamins A, C, and E; carotenoids; free metals such as selenium (Se), Cu, and Zn; nutritional lipids such as polyunsaturated fatty acids (PUFAs); bioactive peptides; and amino acids [68].

Micronutrients include vitamin C, vitamin E, and some minerals, as discussed in the extracellular antioxidant defense section of this review. Vitamin E is lipid-soluble and active in membranes and has been used as a supplement in UC treatments. In addition, minerals such as Zn and Se are essential factors for the antioxidant enzymes SOD and GPX. It has been reported that combined treatments with Zn and vitamin E achieved better effects on redox balance than either treatment alone in a rat model [229].

Carotenoids, which exist in plants, algae, and microbiota, act against oxidative damage, protecting the cell and preventing heavy metal accumulation in the body. Humans and animals can only acquire carotenoids from their diets and cannot manufacture them by themselves. Astaxanthin and fucoxanthin are two major carotenoids in marine animals and plants that exert strong antioxidant effects by scavenging free radicals [230].

Se is essential for the catalytic activity of GSH peroxidase, and Se deficiency directly leads to GSH defects and higher oxidative stress. In clinical trials, Se deficiency and selenoprotein inactivation in CD and UC patients have been associated with disease severity [231]. Se-deficient mice subjected to DSS-induced epithelial injuries show increased tumorigenesis and progression after AOM/DSS treatment [232]. These findings indicate the significance of selenoprotein as potent antioxidants.

Of the PUFAs, omega-3 fatty acids are the most efficient in attenuating inflammation in IBD. Omega-3 fatty acids can be derived from different resources, such as nuts, fish, and linseed. These fatty acids are healthy and can reduce weight loss, decrease inflammation, promote membrane homeostasis, and improve the body's peripheral neural system. Omega-3 fatty acids are also substrates for resolvin, maresin, and protectin synthesis and may interfere with IBD progression by reducing oxidative stress and decreasing the expression of TNF-*α* and inflammatory cytokines. Many clinical and animal experiments have been conducted to explore the benefits of omega-3 fatty acids in IBD. However, their effects remain controversial due to the various forms of omega-3 fatty acids, varied responses to diverse genetic receptors, and differences in methodology. More valid data regarding omega-3 fatty acids are required to determine their clinical applications in IBD [233].

In addition, bioactive peptides and amino acids have been demonstrated to attenuate intestine inflammation, reduce oxidative stress, and recover mucosal homeostasis in IBD models, suggesting another alternative dietary therapy for IBD [234].

### 5.6. Probiotics

Probiotics, living microorganisms that can benefit the host, have attracted increasing research interests in recent years. Probiotics that enhance antioxidant defenses are involved in ROS removal, enzyme inhibition, metal chelation, and antioxidant enzyme synthesis. Some research reports have demonstrated that probiotics can increase SOD, CAT, and GPX activities and GSH levels while decreasing MPO and NO· activities [56, 235].

The most commonly used probiotics are strains of *Lactobacillus* and *Bifidobacterium*, which are reported to secrete enzymes such as SOD and metal-chelating as well as antioxidant molecules such as exopolysaccharides to protect the intestine from IBD or colorectal cancer in rat models [236, 237]. *Lactobacillus* fermentum ME-3 has been reported to improve total antioxidant activity (TAA) and total antioxidant status (TAS) in healthy volunteers demonstrating its probiotic functions against oxidative stress [238]. *E. coli* Nissle 1917, VSL#3 (a food supplement that contains 8 different strains of live lactic acid bacteria), *Lactobacilli*, and *Bifidobacteria* have anti-inflammatory effects and relieve clinical symptoms in UC patients [239]. It has been summarized that the distinctions of disease subtypes, disease location, and medication possibilities as well as the bacterial strains and applied doses should be assessed in future studies to evaluate the use of probiotics in IBD treatments [240].

## 6. Discussion and Future Perspectives

In the current review, we have summarized the current knowledge on oxidative stress in IBD. Increased ROS generation and decreased antioxidant activities contribute to the major pathogenesis and progression of IBD. The GI tract epithelium is continuously exposed to multiple stimuli, such as ingested food, resident microbiota or other infections, gastric acid, and other pro-oxidants [241–244]. During inflammation, immune cells, such as leukocytes, monocytes, and neutrophils augment ROS production during respiratory, prostaglandin, and leukotriene metabolism, resulting in further tissue damages [124]. Several literature reports have found ROS/RNS upregulation in IBD patients [11, 69] as well as in murine colitis models [187, 235, 245]. A significant correlation between oxidative stress and IBD severity has been implied [246]. In addition to overwhelming ROS generation, the defective antioxidant defense is another key factor promoting IBD progression. When antioxidant defenses fail, oxidative stress occurs, which further leads to IBD initiation. In the setting of IBD, downregulation of CAT, GPX, and SOD has been demonstrated in human colonic mucosa, submucosa, and serosa compared with expression in the small intestine. In addition, an overall reduction of nonenzymatic antioxidants such as vitamins, *β*-carotene, tocopherol, and glutathione has been found in IBD patients, and the extent of reduction correlates with the severity of IBD [70, 247–249]. However, with regard to antioxidant levels in clinical trials and animal models of IBD, different studies have demonstrated controversial or even contradictory results possibly due to the use of different animal models, diverse methods, and varied biomarkers. Thus, more precise experimental and clinical studies should be performed to provide further insights into IBD.

Oxidative stress is now regarded as a potential pathogenic and critical factor in the initiation, progression, and severity of IBD, rather than a consequence of chronic inflammation in the intestinal mucosa. Nevertheless, the underlying mechanisms are still far from thoroughly elucidated. Based on the laboratory reports and clinical trials, increasing numbers of antioxidants, such as ROS generation inhibitors, hormones, synthetic substances, polyphenols and herbal plant extracts, functional foods, micronutrients, and probiotics, are potential therapeutic substances that target oxidative stress in IBD. However, further studies are needed to confirm the exact effects of these promising drugs and the suitable doses and administration routes. A combination of new antioxidant-enhancing therapies and conventional medications may achieve better curative effects in IBD and should be explored in future clinical trials.
